# Prion Pathogenesis in the Absence of NLRP3/ASC Inflammasomes

**DOI:** 10.1371/journal.pone.0117208

**Published:** 2015-02-11

**Authors:** Mario Nuvolone, Silvia Sorce, Petra Schwarz, Adriano Aguzzi

**Affiliations:** Institute of Neuropathology, University Hospital of Zurich, Zurich, Switzerland; University of Melbourne, AUSTRALIA

## Abstract

The accumulation of the scrapie prion protein PrP^Sc^, a misfolded conformer of the cellular prion protein PrP^C^, is a crucial feature of prion diseases. In the central nervous system, this process is accompanied by conspicuous microglia activation. The NLRP3 inflammasome is a multi-molecular complex which can sense heterogeneous pathogen-associated molecular patterns and culminates in the activation of caspase 1 and release of IL 1β. The NLRP3 inflammasome was reported to be essential for IL 1β release after *in vitro* exposure to the amyloidogenic peptide PrP^106-126^ and to recombinant PrP fibrils. We therefore studied the role of the NLRP3 inflammasome in a mouse model of prion infection. Upon intracerebral inoculation with scrapie prions (strain RML), mice lacking NLRP3 (*Nlrp3^-/-^*) or the inflammasome adaptor protein ASC (*Pycard^-/-^*) succumbed to scrapie with attack rates and incubation times similar to wild-type mice, and developed the classic histologic and biochemical features of prion diseases. Genetic ablation of NLRP3 or ASC did not significantly impact on brain levels of IL 1β at the terminal stage of disease. Our results exclude a significant role for NLRP3 and ASC in prion pathogenesis and invalidate their claimed potential as therapeutic target against prion diseases.

## Introduction

The transition from the cellular prion protein (PrP^C^) to its misfolded, amyloidogenic conformer (PrP^Sc^), is crucial to the generation of infectious prions [1], and is the central feature of a class of invariably fatal, transmissible neurodegenerative disorders termed prion diseases [2]. The adaptive immune system lacks the ability to mount anti-prion immune responses [3], possibly due to similar immunogenicity between PrP^C^ and PrP^Sc^ and, consequently, to immune tolerance. Conversely, peripherally acquired prions often hijack components of the immune system to replicate in lymphoid tissues before invading the central nervous system [4].

In the central nervous system (CNS), prion diseases are accompanied by microglia activation [5–7]. This activation may have beneficial effects, since microglial engulfment of cerebral apoptotic bodies is an important mechanism of prion clearance [8]. However, there is little understanding of the role of the innate immune system against prions [9], and in particular of the mechanisms by which prions activate microglial cells.

Toll-like receptors (TLRs), key membrane-bound molecules for the recognition of invading microorganisms, play a limited defensive role in prion diseases [10–12]. In addition to TLRs, the innate immune system can detect pathogen- or danger-associated molecular patterns (PAMPs or DAMPs, respectively) through different cytoplasmic proteins, of which the best characterized are the nucleotide-binding oligomerization-domain protein-like receptors (NLRs). Among NLRs, NLRP3 (also termed NALP3 or cryopyrin) can sense a plethora of structurally and chemically heterogeneous PAMPs and DAMPs [13–15], including amyloid β in models of Alzheimer’s disease [16,17]. Upon activation, NLRP3 enables the formation of a multi-protein complex named NLRP3 inflammasome, which involves the polymerization of the adaptor protein ASC and culminates in the activation of caspase-1 and in the proteolytic processing and subsequent secretion of IL-1β and IL-18 [18–20].

Using *in vitro* models based on macrophage/microglia cell lines or primary murine microglia, the NLRP3 inflammasome was found to be essential for IL-1β release upon exposure to aggregates of the PrP^C^-derived PrP^106–126^ amyloidogenic neurotoxic peptide [21,22] or *in vitro* fibrillized recombinant PrP [23]. However, whether the NLRP3 inflammasome can sense *bona fide* prions and significantly impact on prion pathogenesis is presently unknown. Here we set out to investigate the role of the NLRP3 inflammasome in prion disease *in vivo*.

## Materials and Methods

### Ethical statements

Animal care and experimental protocols were all performed in compliance with the Swiss Animal Protection Law and with the “Swiss Ethical Principles and Guidelines for Experiments on Animals”, under the approval of the Veterinary office of the Canton Zurich (animal permits 200/2007, 130/2008, 41/2012, 90/2013). Prion inoculations were performed under methoxyfluorane anesthesia. All efforts were made to minimize animal discomfort and suffering.

**Database search.** Cell-specific patterns of gene expression in mouse brains were derived from RNA sequencing of the transcriptome and splicing database of glia, neurons and vascular cells of the cerebral cortex [24], available through the following website: http://web.stanford.edu/group/barres_lab/brain_rnaseq.html.

Microarray-based dynamic gene expression profiles during prion disease development in different combinations of mouse strains and prion strains were obtained through the Prion Disease DataBase (PDDB) [25,26], through the following website: http://prion.systemsbiology.net/page/Welcome/display.

### Mice

NLRP3-deficicent mice (C57BL/6-*Nlrp3* ^*tm1Ts*c^) [27] and ASC-deficient mice (B6.129-*Pycard* ^*tm1Vm*d^) [28], both on a C57BL/6 background, were kindly provided by Prof. Jurg Tschopp and C57BL/6 mice were purchased from Harlan, Nederland. PrP^C^-deficient mice on a C57BL/6 background (B6.129-*Prnp* ^*tm1Cwe*^)[29,30] were maintained in house. Mice were kept in a conventional hygienic grade facility, housed in groups of 3–10 in conventional type II and type III cages, under a 12 h light/12 h dark cycle (from 7 am to 7 pm) at 22±1°C, with unrestricted access to sterilized food (Kliba No. 3340, Provimi Kliba, Kaiseraugst, Switzerland) and water.

### Genotyping

Tissue biopsies were digested in 10 mM Tris pH 9, 50 mM KCl, 0.5% Tween20, 0.5% NP40, 0.1 mg/mL proteinase K (Roche) and 1 µL of crude digest was employed as template for a PCR reaction using the Red Taq ReadyMix PCR reaction Mix (Sigma) on a GeneAmp PCR System 9700 (Applied Biosystems). For the *Nlrp3* ^*tm1Ts*c^ allele, 0.33 μM of each of the following primers were used (5’→3’): AAG TCG TGC TGC TTC ATG T, TCA AGC TAA GAG AAC TTT CTG and ACA CTC GTC ATC TTC AGC A. For the *Pycard* ^*tm1Vm*d^ allele, 0.33 μM of each of the following primers were used (5’→3’): CTA GTT TGC TGG GGA AAG AAC, CTA AGC ACA GTC ATT GTG AGC TCC and AAG ACA ATA GCA GGC ATG CTG G. For both PCR reactions, amplification was performed using the following conditions: 5 min at 94°C; 40 cycles of 30 sec at 94°C, 30 sec at 59°C and 40 sec at 72°C; 5 min at 72°C.

### Prion inoculations

Mice were injected in the right hemisphere with 30 μl of RML6 (passage 6 of Rocky Mountain Laboratory strain mouse-adapted scrapie prions) containing 30µg, 90 ng, 30 ng or 9 ng total protein. Previous titration experiments performed with wild-type CD1 mice showed that the infectivity titer of the RML6 inoculum is Log LD50 g^-1^: 9.3 +/- 0.9 [31]. Control inoculations were performed using 30 µl of non-infectious brain homogenate from CD-1 mice containing 30 µg or 90 ng of total protein.

After inoculation, mice were initially monitored three times per week. After clinical onset, mice were daily monitored and sacrificed in the presence of clear signs of terminal prion disease by cervical dislocation. One *Pycard* ^*-/-*^ mouse inoculated with 90 ng of prions did not develop any sign of prion disease up to 500 days post inoculation, possibly reflecting technical problems with the inoculation, and was exclude from the survival analysis.

### Western blotting

Twenty percent (w/vol) tissue homogenates were prepared in 0.25 M sucrose in PBS using a Ribolyzer (Bio-Rad). Total protein concentration was measured with the BCA Protein Assay (Pierce), according to the manufacturer’s instructions. For the detection of partially PK-resistant PrP, proteins were digested with PK (Roche) at a final concentration of 25 µg/ml. Protein homogenates in NuPAGE LDS sample buffer (Invitrogen) containing β-mercaptoethanol as reducing agent were separated on a NuPAGE 12% Bis-Tris (Invitrogen) using the NuPAGE Gel Electrophoresis System (Invitrogen) and transferred onto a Protran Nitrocellulose Transfer Membrane (Whatman) using the Wet/Tank Blotting System (Bio-Rad), according to the manufacturers’ instructions. Antibodies used were POM1 (200 ng ml–1 [32]) as primary and HRP-conjugated goat anti-mouse IgG (H+L) (1:17000 dilution, from Invitrogen) as secondary. Blots were developed using HRP substrate (ECL, Pierce) and visualized using the Versadoc 3000 imaging system (Bio-Rad).

### ELISA

PrP^C^ was quantified in brain tissue homogenate by POM1/POM2 sandwich ELISA as previously described [32]. IL-1β was quantified in brain tissue homogenate using the Mouse IL-1β ELISA kit (eBioscience) according to manufacturer’s instructions.

### Histology and immunohistochemistry

Stainings were performed on 2 µm sections from formalin fixed, formic acid treated, paraffin embedded tissues. After deparaffinization through graded alcohols, heat-induced antigen retrieval was performed in EDTA-based buffer CC1 and stainings were performed on a NEXES immunohistochemistry robot (Ventana instruments) using the following antibodies: Iba1 (1:1000, Wako); GFAP (1:13000, Dako); SAF84 (1:200, SPI bio). Only for the latter staining, antibody incubation was preceded by incubation with protease 2 (Ventana). Immunoreactivity was visualized using an IVIEW DAB Detection Kit (Ventana). Haematoxylin and eosin staining was performed according to standard procedures. Slides were scanned with NanoZoomer and images were visualized using the NanoZoomer Digital Pathology System (NDPview, Hamamatsu Photonics).

### Statistical analysis

Statistical significance was assessed using GraphPad Prism software with One-way ANOVA and Bonferroni’s multiple comparison post-test, or unpaired Student’s t-test, as appropriate. Kaplan-Meier method was employed to analyse survival times and comparison between genotypes was made with the log-rank test. Alpha level was set at 0.05.

For each statistical analysis, the statistical test, p-value, and the group size are indicated in the corresponding figure legends.

## Results

### Levels of NLRP3 inflammasome-related transcripts during prion disease

We first investigated the pattern of cellular expression of NLRP3 inflammasome components within the mouse brain. For this purpose, we took advantage of the recently published RNA-sequencing-based transcriptome database for mouse brain cortex cells [24]. Transcripts of *Nlrp3*, *Pycard*, *Casp1* and *Il1b* genes, encoding NLRP3, ASC, caspase 1 and IL-1β, respectively, showed a prevalent expression in microglial cells (S1A Fig.).

Next, we studied temporal changes of NLRP3 inflammasome-related transcripts in the brain during prion disease development. To this aim, we interrogated the prion disease database, which includes microarray-based gene expression data in mouse models of prion disease [25,26]. We analysed different time points during prion disease and different combinations of both mouse and prion strains [25,26]. We found that these transcripts are either unchanged (*Nlrp3* and *Il1b*) or only moderately upregulated (*Pycard* and, to a less extent, *Casp1*) in the latest stages of prion disease with respect to baseline levels (S1B Fig.). These changes were modest in comparison to other microglia specific transcripts, as *Cd68* (S1B Fig.). Overall these data indicate that NLRP3 inflammasome-related transcripts are only marginally affected during prion disease.

### NLRP3 and prion pathogenesis

**Fig 1:**
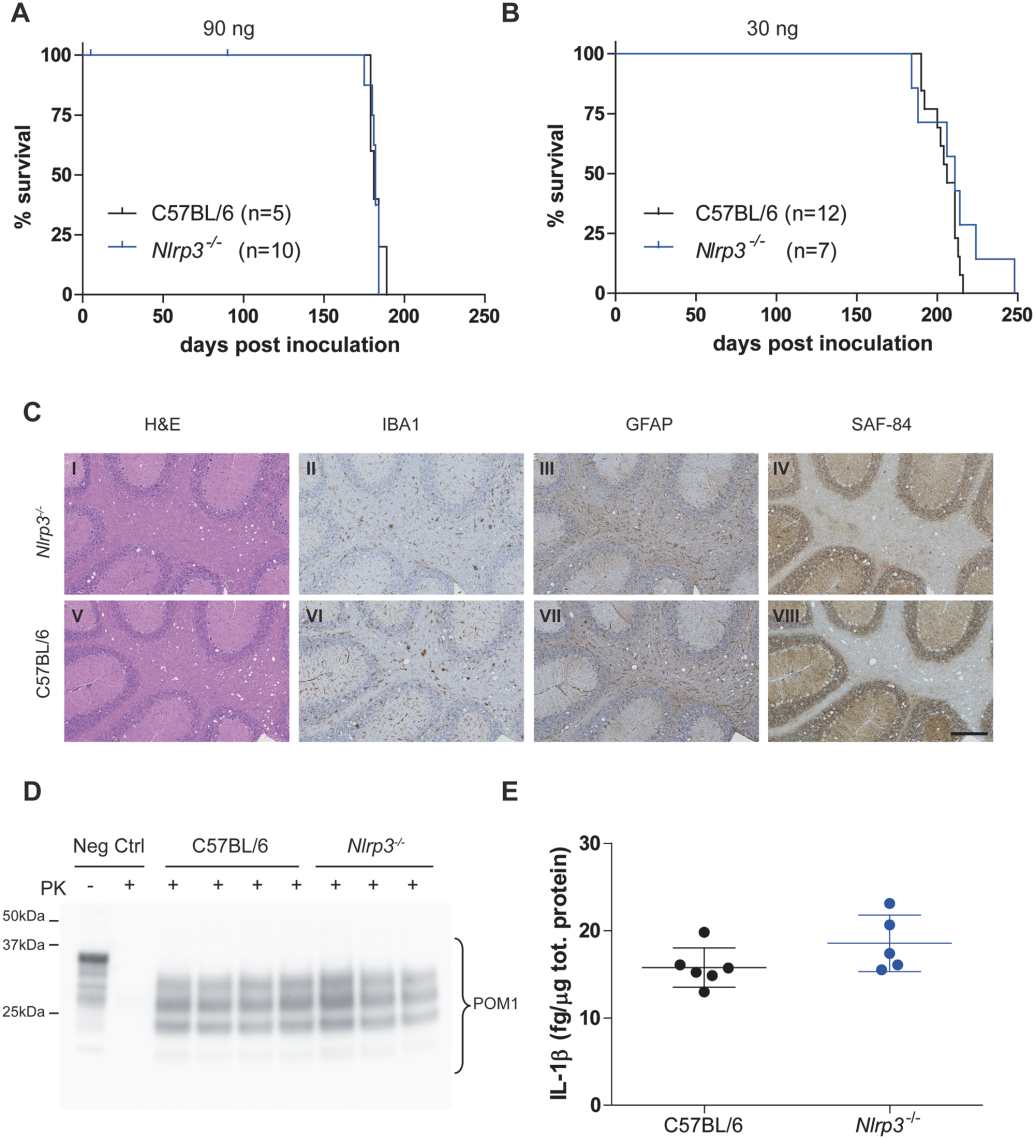
Prion disease in the absence of NLRP3. **A-B** Kaplan-Meier survival plots of *Nlrp3* ^*-/-*^ (blue line) and C57BL/6 wild-type mice (black line) inoculated intracerebrally with 90 ng (A) or 30 ng of RML6 (B). For each experimental group, the number of mice is indicated (n). Censored events (ticks) indicate intercurrent deaths not related with prion disease. With both doses, no statistically significant difference was observed (90 ng = median survival *Nlrp3* ^*-/-*^ 182 dpi, C57BL/6 181 dpi, P = 0.79; 30 ng = *Nlrp3* ^*-/-*^ 211 dpi, C57BL/6 206 dpi, P = 0.21, log-rank test). **C** Histological analysis of *Nlrp3* ^*-/-*^ (I-IV) and C57BL/6 wild-type mice (V-VIII). Representative hematoxylin and eosin staining (H&E) and immunohistochemical stainings for microglia (IBA1), astrocytes (GFAP) and partially protease-resistant PrP (SAF-84) in cerebellar areas are displayed. Scale bar: 250 µm. **D** Western blotting analysis of partially protease K (PK)-resistant PrP as detected with POM1 in brain homogenates after PK digestion (PK +). Neg Ctrl: brain homogenate from a control mouse not inoculated with prions is shown with (PK +) and without (PK -) PK digestion. **E** Levels of IL-1β in brains of terminally sick mice. Each point denotes one mouse. Mean +/- standard deviation are shown. No significant difference was observed (P = 0.13, Student’s t test). C, D and E are referred to mice inoculated with 30 ng of RML6.

To investigate the role of NLRP3 in prion pathogenesis, we elected to use NLRP3-deficient (*Nlrp3* ^*-/-*^) mice [27]. We first verified that genetic ablation of NLRP3 did not significantly influence PrP^C^ levels. Sandwich ELISA on brain homogenates from *Nlrp3* ^*-/-*^ and C57BL/6 wild-type mice excluded such an effect (S2 Fig.). We then injected intracerebrally (i.c.) *Nlrp3* ^*-/-*^ and C57BL/6 wild-type mice with mouse-adapted scrapie prions (Rocky Mountain Laboratory strain, passage #6; RML6) [31]. *Nlrp3* ^*-/-*^ and C57BL/6 wild-type mice displayed similar survival times after inoculation with two different doses of RML6 prions (Fig. 1A-B; 90 ng = median survival *Nlrp3* ^*-/-*^ 182 dpi, C57BL/6 181 dpi; P = 0.79, log-rank test; 30 ng = *Nlrp3* ^*-/-*^ 211 dpi, C57BL/6 206 dpi; P = 0.21, log-rank test). Both genotypes showed histologic features typical of prion disease, including spongiosis, micro- and astrogliosis as well as accumulation of partially PK-resistant PrP in brains as assessed by Western blotting (Fig. 1C-D). Conversely, upon i.c. injection of 90 ng of non-infectious brain homogenate, *Nlrp3* ^*-/-*^ mice survived more than 450 days in the absence of clinical signs of prion disease (S3 Fig.). We next asked whether NLRP3 genetic ablation would have an impact on the production of IL-1β in brains of prion-infected mice. We measured IL-1β levels in brains of terminally sick mice that received 30 ng of RML6 i.c. by sandwich ELISA. We saw no significant difference in IL-1β levels between *Nlrp3* ^*-/-*^ and C57BL/6 control brains (Fig. 1E). Brain IL-1β levels also did not differ between terminally sick C57BL/6 mice and control mice injected with non-infectious brain homogenate (S4 Fig.). These results exclude a significant effect of NLRP3 genetic ablation on prion occurrence and incubation as well as on brain IL-1β levels at the terminal stage of the disease.

### ASC and prion pathogenesis

**Fig 2:**
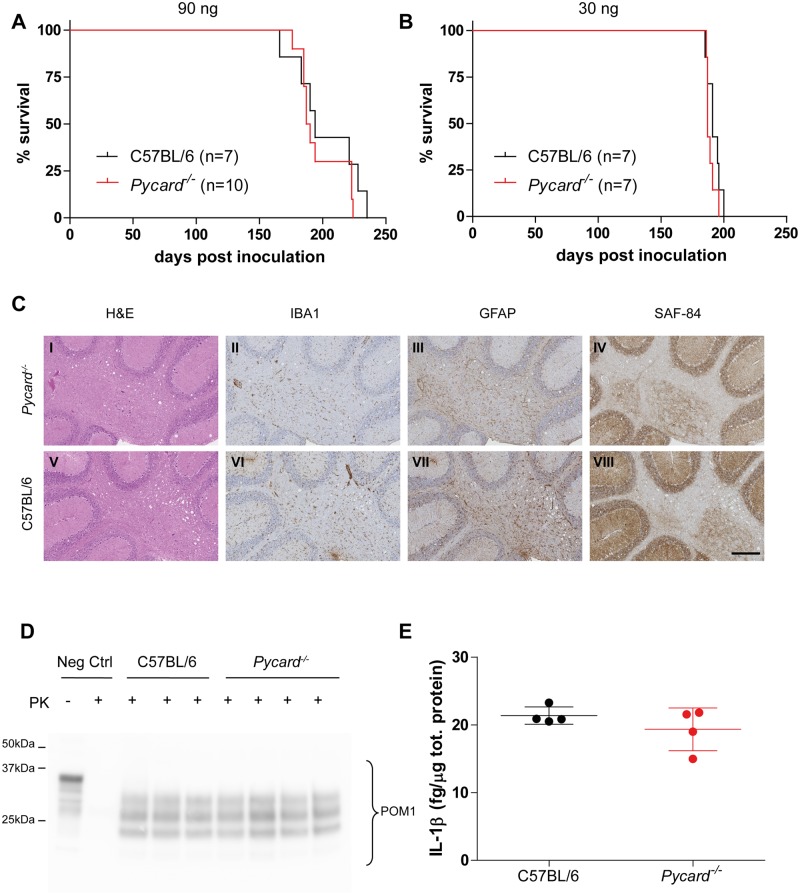
Prion disease in the absence of ASC. **A-B** Kaplan-Meier survival plots of *Pycard* ^*-/-*^ (red line) and C57BL/6 wild-type mice (black line) inoculated intracerebrally with 90 ng (A) or 30 ng of RML6 prions (B). For each experimental group, the number of mice is indicated (n). With both doses, no statistically significant difference was observed (90 ng = median survival *Pycard* ^*-/-*^ 189 dpi, C57BL/6 194 dpi, P = 0.35; 30 ng = *Pycard* ^*-/-*^ 187 dpi, C57BL/6 191 dpi, P = 0.23, log-rank test). **C** Histological analysis of *Pycard* ^*-/-*^ (I-IV) and C57BL/6 wild-type mice (V-VIII). Representative hematoxylin and eosin staining (H&E) and immunohistochemical stainings for microglia (IBA1), astrocytes (GFAP) and partially protease-resistant PrP (SAF-84) in cerebellar areas are displayed. Scale bar: 250 µm. **D** Western blotting analysis of partially protease K (PK)-resistant PrP as detected with POM1 in brain homogenates after PK digestion (PK +). Neg Ctrl: brain homogenate from a control mouse not inoculated with prions is shown with (PK +) and without (PK -) PK digestion. **E** Levels of IL-1β in brains of terminally sick mice. Each point denotes one mouse. Mean +/- standard deviation are shown. No significant difference was observed (P = 0.28, Student’s t test). C, D and E are referred to mice inoculated with 30 ng of RML6.

To further validate our results, we investigated the effect of genetic ablation of the NLRP3 inflammasome adaptor protein ASC on prion disease using ASC-deficient (*Pycard* ^*-/-*^) mice [28]. Again, genetic ablation of ASC did not alter brain PrP^C^ levels (S2 Fig.). After i.c. inoculation with RML6 prions, both *Pycard* ^*-/-*^ and C57BL/6 wild-type mice presented similar survivals (Fig. 2A-B; 90 ng = median survival *Pycard* ^*-/-*^ 189 dpi, C57BL/6 194 dpi; P = 0.35, log-rank test; 30 ng = *Pycard* ^*-/-*^ 187 dpi, C57BL/6 191 dpi; P = 0.23, log-rank test). Both genotypes showed histologic features characteristic of prion disease, accumulation of partially PK-resistant PrP and similar levels of IL-1β in the brain (Fig. 2 C-E). *Pycard* ^*-/-*^ mice injected i.c. with 90 ng of normal brain homogenate did not develop any sign of prion disease and survived more than 450 days post-injection (S3 Fig.).

When using a lower dose of prions (9 ng of RML6 i.c.), *Nlrp3* ^*-/-*^, *Pycard* ^*-/-*^ and C57BL/6 wild-type mice showed an incomplete attack rate (11/16, 8/13, 7/15, respectively), with no significant difference in survival among the three genotypes (S3 Fig.). These results rule out a significant effect of ASC genetic ablation on prion occurrence and incubation as well as on brain IL-1β levels at the terminal stage of the disease.

## Discussion

Here we have investigated the role of the NLRP3 inflammasome on prion disease *in vivo*. Genetic ablation of NLRP3 did not exert any significant impact on prion pathogenesis upon i.c. inoculation with different doses of RML6 prions. NLRP3-deficient mice showed similar incubation times compared to wild-type controls and displayed biochemical and neuropathological features characteristic of prion diseases. These data exclude a significant role for NLRP3 inflammasome in our model.

Similar results were obtained with mice lacking the adaptor protein ASC, confirming our results with *Nlrp3* ^*-/-*^ mice and incontrovertibly ruling out any requirement for several NLRP3-independent, ASC-dependent inflammasomes, including the AIM2 [33], NLRP6 [34] and NLRP12 [35] inflammasomes and the non-canonical MALT1-ASC-caspase 8 inflammasome [36]. Also, even if NLRP1 and NLRC4 can induce caspase-1 activation and IL-1β production independently of ASC, it has been shown that ASC is required for the optimal activation of NLRP1 [37] and NLRC4 [38] inflammasomes.

The IL-1β response to prions is the subject of controversies. Increased levels of IL-1β have been reported in the cerebrospinal fluid of patients affected by Creutzfeldt-Jakob disease [39,40], as well as in the brain of mice or hamsters inoculated with rodent-adapted scrapie prions [41–43]. However, other reports failed to confirm these observations [44,45]. The source of this increase in IL-1β during prion diseases has not been elucidated. Recently, Heneka and coworkers reported that genetic ablation of NLRP3 resulted in a significant reduction of total IL-1β levels in brains of transgenic mice overexpressing human amyloid β (APPPS1 *NLRP3* ^*-/-*^ vs APPPS1), whereas brain levels of IL-1β were not different between wild-type and *NLRP3* ^*-/-*^ mice [17].

This controversial state of affairs motivated us to investigate the contribution of NLRP3/ASC inflammasomes to brain levels of IL-1β in our experimental model of prion disease. We found no significant difference in total IL-1β levels in brains of terminally sick mice inoculated with RML6 prions as compared to brains of mice injected with non-infectious brain homogenate. Also, contrary to what might have been predicted from the findings discussed above, we found that genetic ablation of NLRP3 or ASC did not significantly alter the brain levels of IL-1β at the terminal stage of the disease. This result does not necessarily contradict previous studies showing the importance of NLRP3/ASC inflammasomes for IL-1β production [13–15,18,46], *in vitro* and *in vivo*, in light of the existence of alternative mechanisms leading to IL-1β secretion independently of NLRP3/ASC inflammasomes. These mechanisms can rely on other NOD-like receptor, like NOD1 [47] or on extracellular proteases, including proteinase 3 [48,49], matrix metalloproteinases [50], mast cell chymase [51] and others. In particular, several IL-1β-mediated phenotypes have been reported to be unaltered in the absence of caspase-1, underscoring the relevance of additional sources of IL-1β independent of NLRP3/ASC inflammasomes [52,53]. It is possible that some of these mechanisms may be of relevance to prion pathogenesis. Interestingly, several studies showed an upregulation of IL-1β transcripts in mouse scrapie [54–57], in naturally-occurring scrapie of sheep [58] and in human Creutzfeldt-Jakob disease cases [59,60]. Collectively, these data indicate that transcriptional mechanisms could be of relevance for the release of IL-1β during prion disease. On the other hand, other studies failed to detect a significant change in IL-1β transcripts during prion disease development [25,26]. Again, the origin of these discrepancies has yet to be established.

Mice lacking the IL-1 type 1 receptor (IL-1R1) were reported to have an increased survival compared to wild-type controls after prion inoculation [57,61], but it remained unclear whether this effect was due to the lack of IL-1β signaling through the IL-1R1 receptor. IL-1α is constitutively expressed by a variety of cells, including astrocytes within the CNS, also binds IL-1R1, and it has biological properties similar to IL-1β [46]. The role of IL-1α in prion pathogenesis is presently unknown.

The NLRP3 inflammasome was claimed to be crucially implicated in the release of IL-1β upon exposure to the amyloidogenic peptide PrP^106–126^ [21,22] and to recombinant PrP fibrils [23], at least *in vitro*. This process was dependent on phagocytosis of PrP-derived moieties and subsequent lysosomal destabilization [23,62], reminiscent of the mechanism underlying NLRP3 inflammasome-dependent release of IL-1β caused by amyloid β [16,17]. Based on these studies, it was postulated that pharmacological interference with the NLRP3 inflammasome/IL-1β signaling pathway could be beneficial against prion diseases [21,23,62]. However, in our study the genetic ablation of NLRP3 or ASC did not ameliorate the course of the disease. There have been previous examples of incongruences between promising *in vitro* studies reporting activation of NLRP3 inflammasome by a specific pathogen and disappointing *in vivo* investigations of the same pathogen, for example for *Mycobacterium tuberculosis* [63,64]. These discrepancies can reflect the more complex situation of *in vivo* vs. *in vitro* experimental paradigms. In the case of prion disorders, all previous studies suggesting a role for the NLRP3 inflammasomes were conducted with the PrP^106–126^ neurotoxic peptides or *in vitro* generated PrP aggregates or fibrils which, unlike *bona fide* prions, are not infectious and may trigger neurotoxicity by different pathways from those activated in prion infections.

In our study, mice lacking NLRP3 or ASC were inoculated with rodent-adapted scrapie prions of the RML type. However prions exist in many different strains differing for their structural properties and causing disease with specific features when inoculated into genetically identical hosts [65]. Hafner-Bratkovic et al. reported that the NLRP3 inflammasome was activated only by PrP fibrils and large aggregates, but not by oligomers and monomers, suggesting the size of PrP aggregates as an important determinant for this phenomenon [23]. The size of the most infectious prion particles as well as the dynamics of prion replication and processing in different cells of the CNS are all strain-dependent properties of the infectious agent [66,67]. Also, microglia activation and brain levels of inflammatory cytokines have been reported to diverge in different types of prion diseases, possibly reflecting strain-specific features [68–70]. Based on these considerations, it is not excluded that an involvement of NLRP3 or ASC may become more apparent in specific forms of prion diseases different from those studied here.

Be as it may, our study adds to the general consensus that the identification of realistic therapeutic targets necessitates robust animal models which recapitulate the salient aspects of the disease in question. Failure to adhere to the latter has led research astray in a worrisome number of cases [71]. Once again, we found that a claim of therapeutic efficacy solely based on exposure of cell lines to an artificial aggregate such as the amyloidogenic peptide PrP^106–126^, which bears little resemblance to *bona fide* prions, can lead to unjustified and essentially incorrect conclusions.

## Supporting Information

S1 ARRIVE Checklist Checklist according to ARRIVE guidelines for Reporting Animal Research—*In vivo* experiments. (PDF)

S1 Fig NLRP3 inflammasome-related transcripts during prion disease. **A** Expression levels of different NLRP3 inflammasome-related transcripts in different cell types of mouse brain cortex as assessed by RNA-sequencing of acutely purified cell populations. OPC: oligodendrocyte precursor cells; FPKM: fragments per kilobase of transcript per million mapped reads. Based on whole transcriptome profile, it is concluded that OPC cell preparation has 5% of microglia contamination. Scale bars indicate standard deviation. Data and graphs are from Zhang et al. J Neurosci 2014 [24]. **B** Dynamic gene expression profiles during prion disease development in four mouse strain → prion strain combinations as obtained by microarray analysis. RML→ FVB.129-Prnp0/0 combination serves as control, as these mice lack PrPC and are resistant to prion infection. Data are from Hwang et al. Mol Systm Biol 2009 [25,26].
(TIF)

S2 Fig PrP^C^ levels in CNS of mice lacking NLRP3/ASC. **A-B** Levels of PrPC in forebrain (A) and cerebellum (B) of Nlrp3-/-, Pycard-/- and C57BL/6 wild-type control mice. Each point denotes one mouse. Mean +/- standard deviation are shown. No significant difference was observed (P = 0.24 in A, P = 0.15 in B, One-way ANOVA).
(TIF)

S3 Fig Brain IL-1β levels in terminally prion infected and control injected mice. Levels of IL-1β in brains of terminally sick C57BL/6 mice (RML) and control mice injected with non-infectious brain homogenate (NBH). Mice received 30 µg of RML6 or control homogenate. Each point denotes one mouse. Mean +/- standard deviation are shown. No significant difference was observed (P = 0.40, Student’s t test).
(TIF)

S4 Fig Survival upon intracerebral inoculation with a low dose of prions. Kaplan-Meier survival plots of Nlrp3^-/-^ (blue line), Pycard^-/-^ (red line) and C57BL/6 wild-type mice (black line) inoculated intracerebrally with 9 ng of RML6. No statistically significant difference among prion-inoculated mice was observed (attack rate and median survival: Nlrp3^-/-^, 11/16, 241 dpi; Pycard^-/-^ 8/13, 256 dpi; C57BL/6, 7/15, median survival not reached; P = 0.55, log-rank test). Dashed lines indicate mice injected with 90 ng of non-infectious brain homogenates (NBH). For each experimental group, the number of mice is indicated (n). Censored events (ticks) indicate intercurrent deaths not related with prion disease or termination of the experiment.
(TIF)

S5 Fig Uncropped images of Western blots presented in this study. **A** Original image of Western blot presented in Fig. 1D and the relative image showing molecular size marker in **B**. **C** Original image of Western blot presented in Fig. 2D and the relative image showing molecular size marker in **D**.
(TIF)

## References

[1] Prusiner SB (1982) Novel proteinaceous infectious particles cause scrapie. Science 216: 136–144. 6801762 10.1126/science.6801762

[2] Aguzzi A, Calella AM (2009) Prions: protein aggregation and infectious diseases. Physiol Rev 89: 1105–1152. 10.1152/physrev.00006.2009 19789378

[3] Polymenidou M, Heppner FL, Pellicioli EC, Urich E, Miele G, et al (2004) Humoral immune response to native eukaryotic prion protein correlates with anti-prion protection. Proc Natl Acad Sci U S A 101 Suppl 2: 14670–14676. 15292505 10.1073/pnas.0404772101 PMC521983

[4] Aguzzi A, Nuvolone M, Zhu C (2013) The immunobiology of prion diseases. Nat Rev Immunol 13: 888–902. 10.1038/nri3553 24189576

[5] Barcikowska M, Liberski PP, Boellaard JW, Brown P, Gajdusek DC, et al (1993) Microglia is a component of the prion protein amyloid plaque in the Gerstmann-Straussler-Scheinker syndrome. Acta Neuropathol 85: 623–627. 8337941 10.1007/BF00334672

[6] Guiroy DC, Wakayama I, Liberski PP, Gajdusek DC (1994) Relationship of microglia and scrapie amyloid-immunoreactive plaques in kuru, Creutzfeldt-Jakob disease and Gerstmann-Straussler syndrome. Acta Neuropathol 87: 526–530. 8059606 10.1007/BF00294180

[7] Williams A, Lucassen PJ, Ritchie D, Bruce M (1997) PrP deposition, microglial activation, and neuronal apoptosis in murine scrapie. Exp Neurol 144: 433–438. 9168844 10.1006/exnr.1997.6424

[8] Kranich J, Krautler NJ, Falsig J, Ballmer B, Li S, et al (2010) Engulfment of cerebral apoptotic bodies controls the course of prion disease in a mouse strain-dependent manner. J Exp Med 207: 2271–2281. 10.1084/jem.20092401 20837697 PMC2947076

[9] Bradford BM, Mabbott NA (2012) Prion disease and the innate immune system. Viruses 4: 3389–3419. 23342365 10.3390/v4123389 PMC3528271

[10] Prinz M, Heikenwalder M, Schwarz P, Takeda K, Akira S, et al (2003) Prion pathogenesis in the absence of Toll-like receptor signalling. EMBO Rep 4: 195–199. 12612611 10.1038/sj.embor.embor731 PMC1315829

[11] Spinner DS, Cho IS, Park SY, Kim JI, Meeker HC, et al (2008) Accelerated prion disease pathogenesis in Toll-like receptor 4 signaling-mutant mice. J Virol 82: 10701–10708. 10.1128/JVI.00522-08 18715916 PMC2573175

[12] Ishibashi D, Atarashi R, Fuse T, Nakagaki T, Yamaguchi N, et al (2012) Protective role of interferon regulatory factor 3-mediated signaling against prion infection. J Virol 86: 4947–4955. 10.1128/JVI.06326-11 22379081 PMC3347345

[13] Schroder K, Tschopp J (2010) The inflammasomes. Cell 140: 821–832. 10.1016/j.cell.2010.01.040 20303873

[14] Strowig T, Henao-Mejia J, Elinav E, Flavell R (2012) Inflammasomes in health and disease. Nature 481: 278–286. 10.1038/nature10759 22258606

[15] Lamkanfi M, Dixit VM (2014) Mechanisms and functions of inflammasomes. Cell 157: 1013–1022. 10.1016/j.cell.2014.04.007 24855941

[16] Halle A, Hornung V, Petzold GC, Stewart CR, Monks BG, et al (2008) The NALP3 inflammasome is involved in the innate immune response to amyloid-beta. Nat Immunol 9: 857–865. 10.1038/ni.1636 18604209 PMC3101478

[17] Heneka MT, Kummer MP, Stutz A, Delekate A, Schwartz S, et al (2013) NLRP3 is activated in Alzheimer’s disease and contributes to pathology in APP/PS1 mice. Nature 493: 674–678. 10.1038/nature11729 23254930 PMC3812809

[18] Agostini L, Martinon F, Burns K, McDermott MF, Hawkins PN, et al (2004) NALP3 forms an IL-1beta-processing inflammasome with increased activity in Muckle-Wells autoinflammatory disorder. Immunity 20: 319–325. 15030775 10.1016/s1074-7613(04)00046-9

[19] Franklin BS, Bossaller L, De Nardo D, Ratter JM, Stutz A, et al (2014) The adaptor ASC has extracellular and 'prionoid' activities that propagate inflammation. Nat Immunol 15: 727–737. 10.1038/ni.2913 24952505 PMC4116676

[20] Baroja-Mazo A, Martin-Sanchez F, Gomez AI, Martinez CM, Amores-Iniesta J, et al (2014) The NLRP3 inflammasome is released as a particulate danger signal that amplifies the inflammatory response. Nat Immunol 15: 738–748. 10.1038/ni.2919 24952504

[21] Shi F, Yang L, Kouadir M, Yang Y, Wang J, et al (2012) The NALP3 inflammasome is involved in neurotoxic prion peptide-induced microglial activation. J Neuroinflammation 9: 73 10.1186/1742-2094-9-73 22531291 PMC3394218

[22] Chang J, Yang L, Kouadir M, Peng Y, Zhang S, et al (2012) Antibody-mediated inhibition of integrin alpha5beta1 blocks neurotoxic prion peptide PrP106–126-induced activation of BV2 microglia. J Mol Neurosci 48: 248–252. 10.1007/s12031-012-9821-6 22648512

[23] Hafner-Bratkovic I, Bencina M, Fitzgerald KA, Golenbock D, Jerala R (2012) NLRP3 inflammasome activation in macrophage cell lines by prion protein fibrils as the source of IL-1beta and neuronal toxicity. Cell Mol Life Sci 69: 4215–4228. 10.1007/s00018-012-1140-0 22926439 PMC3508391

[24] Zhang Y, Chen K, Sloan SA, Bennett ML, Scholze AR, et al (2014) An RNA-sequencing transcriptome and splicing database of glia, neurons, and vascular cells of the cerebral cortex. J Neurosci 34: 11929–11947. 10.1523/JNEUROSCI.1860-14.2014 25186741 PMC4152602

[25] Hwang D, Lee IY, Yoo H, Gehlenborg N, Cho JH, et al (2009) A systems approach to prion disease. Mol Syst Biol 5: 252 10.1038/msb.2009.10 19308092 PMC2671916

[26] Gehlenborg N, Hwang D, Lee IY, Yoo H, Baxter D, et al (2009) The Prion Disease Database: a comprehensive transcriptome resource for systems biology research in prion diseases . Database (Oxford) 2009: bap011. 20157484 10.1093/database/bap011 PMC2790306

[27] Martinon F, Petrilli V, Mayor A, Tardivel A, Tschopp J (2006) Gout-associated uric acid crystals activate the NALP3 inflammasome. Nature 440: 237–241. 16407889 10.1038/nature04516

[28] Mariathasan S, Weiss DS, Newton K, McBride J, O'Rourke K, et al (2006) Cryopyrin activates the inflammasome in response to toxins and ATP. Nature 440: 228–232. 16407890 10.1038/nature04515

[29] Bueler H, Fischer M, Lang Y, Bluethmann H, Lipp HP, et al (1992) Normal development and behaviour of mice lacking the neuronal cell-surface PrP protein. Nature 356: 577–582. 1373228 10.1038/356577a0

[30] Nuvolone M, Kana V, Hutter G, Sakata D, Mortin-Toth SM, et al (2013) SIRPalpha polymorphisms, but not the prion protein, control phagocytosis of apoptotic cells. J Exp Med 210: 2539–2552. 10.1084/jem.20131274 24145514 PMC3832919

[31] Falsig J, Julius C, Margalith I, Schwarz P, Heppner FL, et al (2008) A versatile prion replication assay in organotypic brain slices. Nat Neurosci 11: 109–117. 18066056 10.1038/nn2028 PMC2754795

[32] Polymenidou M, Moos R, Scott M, Sigurdson C, Shi YZ, et al (2008) The POM monoclonals: a comprehensive set of antibodies to non-overlapping prion protein epitopes. PLoS One 3: e3872 10.1371/journal.pone.0003872 19060956 PMC2592702

[33] Hornung V, Ablasser A, Charrel-Dennis M, Bauernfeind F, Horvath G, et al (2009) AIM2 recognizes cytosolic dsDNA and forms a caspase-1-activating inflammasome with ASC. Nature 458: 514–518. 10.1038/nature07725 19158675 PMC2726264

[34] Grenier JM, Wang L, Manji GA, Huang WJ, Al-Garawi A, et al (2002) Functional screening of five PYPAF family members identifies PYPAF5 as a novel regulator of NF-kappaB and caspase-1. FEBS Lett 530: 73–78. 12387869 10.1016/s0014-5793(02)03416-6

[35] Wang L, Manji GA, Grenier JM, Al-Garawi A, Merriam S, et al (2002) PYPAF7, a novel PYRIN-containing Apaf1-like protein that regulates activation of NF-kappa B and caspase-1-dependent cytokine processing. J Biol Chem 277: 29874–29880. 12019269 10.1074/jbc.M203915200

[36] Gringhuis SI, Kaptein TM, Wevers BA, Theelen B, van der Vlist M, et al (2012) Dectin-1 is an extracellular pathogen sensor for the induction and processing of IL-1beta via a noncanonical caspase-8 inflammasome. Nat Immunol 13: 246–254. 10.1038/ni.2222 22267217

[37] Faustin B, Lartigue L, Bruey JM, Luciano F, Sergienko E, et al (2007) Reconstituted NALP1 inflammasome reveals two-step mechanism of caspase-1 activation. Mol Cell 25: 713–724. 17349957 10.1016/j.molcel.2007.01.032

[38] Case CL, Roy CR (2011) Asc modulates the function of NLRC4 in response to infection of macrophages by Legionella pneumophila. MBio 2 10.1128/mBio.00280-11 21771913 PMC3269931

[39] Van Everbroeck B, Dewulf E, Pals P, Lubke U, Martin JJ, et al (2002) The role of cytokines, astrocytes, microglia and apoptosis in Creutzfeldt-Jakob disease. Neurobiol Aging 23: 59–64. 11755020 10.1016/s0197-4580(01)00236-6

[40] Sharief MK, Green A, Dick JP, Gawler J, Thompson EJ (1999) Heightened intrathecal release of proinflammatory cytokines in Creutzfeldt-Jakob disease. Neurology 52: 1289–1291. 10214763 10.1212/wnl.52.6.1289

[41] Williams A, Van Dam AM, Ritchie D, Eikelenboom P, Fraser H (1997) Immunocytochemical appearance of cytokines, prostaglandin E2 and lipocortin-1 in the CNS during the incubation period of murine scrapie correlates with progressive PrP accumulations. Brain Res 754: 171–180. 9134973 10.1016/s0006-8993(97)00067-x

[42] Tribouillard-Tanvier D, Race B, Striebel JF, Carroll JA, Phillips K, et al (2012) Early cytokine elevation, PrPres deposition, and gliosis in mouse scrapie: no effect on disease by deletion of cytokine genes IL-12p40 and IL-12p35. J Virol 86: 10377–10383. 10.1128/JVI.01340-12 22787236 PMC3457249

[43] Xie WL, Shi Q, Zhang J, Zhang BY, Gong HS, et al (2013) Abnormal activation of microglia accompanied with disrupted CX3CR1/CX3CL1 pathway in the brains of the hamsters infected with scrapie agent 263K. J Mol Neurosci 51: 919–932. 10.1007/s12031-013-0002-z 23526370

[44] Stoeck K, Bodemer M, Zerr I (2006) Pro- and anti-inflammatory cytokines in the CSF of patients with Creutzfeldt-Jakob disease. J Neuroimmunol 172: 175–181. 16330103 10.1016/j.jneuroim.2005.10.008

[45] Walsh DT, Betmouni S, Perry VH (2001) Absence of detectable IL-1beta production in murine prion disease: a model of chronic neurodegeneration. J Neuropathol Exp Neurol 60: 173–182. 11273005 10.1093/jnen/60.2.173

[46] Garlanda C, Dinarello CA, Mantovani A (2013) The interleukin-1 family: back to the future. Immunity 39: 1003–1018. 10.1016/j.immuni.2013.11.010 24332029 PMC3933951

[47] Kavathas PB, Boeras CM, Mulla MJ, Abrahams VM (2013) Nod1, but not the ASC inflammasome, contributes to induction of IL-1beta secretion in human trophoblasts after sensing of Chlamydia trachomatis. Mucosal Immunol 6: 235–243. 10.1038/mi.2012.63 22763410 PMC3465624

[48] Coeshott C, Ohnemus C, Pilyavskaya A, Ross S, Wieczorek M, et al (1999) Converting enzyme-independent release of tumor necrosis factor alpha and IL-1beta from a stimulated human monocytic cell line in the presence of activated neutrophils or purified proteinase 3. Proc Natl Acad Sci U S A 96: 6261–6266. 10339575 10.1073/pnas.96.11.6261 PMC26869

[49] Joosten LA, Netea MG, Fantuzzi G, Koenders MI, Helsen MM, et al (2009) Inflammatory arthritis in caspase 1 gene-deficient mice: contribution of proteinase 3 to caspase 1-independent production of bioactive interleukin-1beta. Arthritis Rheum 60: 3651–3662. 10.1002/art.25006 19950280 PMC2993325

[50] Schonbeck U, Mach F, Libby P (1998) Generation of biologically active IL-1 beta by matrix metalloproteinases: a novel caspase-1-independent pathway of IL-1 beta processing. J Immunol 161: 3340–3346. 9759850

[51] Mizutani H, Schechter N, Lazarus G, Black RA, Kupper TS (1991) Rapid and specific conversion of precursor interleukin 1 beta (IL-1 beta) to an active IL-1 species by human mast cell chymase. J Exp Med 174: 821–825. 1919436 10.1084/jem.174.4.821 PMC2118970

[52] Fantuzzi G, Ku G, Harding MW, Livingston DJ, Sipe JD, et al (1997) Response to local inflammation of IL-1 beta-converting enzyme- deficient mice. J Immunol 158: 1818–1824. 9029121

[53] Cheng W, Shivshankar P, Li Z, Chen L, Yeh IT, et al (2008) Caspase-1 contributes to Chlamydia trachomatis-induced upper urogenital tract inflammatory pathologies without affecting the course of infection. Infect Immun 76: 515–522. 18025098 10.1128/IAI.01064-07 PMC2223466

[54] Campbell IL, Eddleston M, Kemper P, Oldstone MB, Hobbs MV (1994) Activation of cerebral cytokine gene expression and its correlation with onset of reactive astrocyte and acute-phase response gene expression in scrapie. J Virol 68: 2383–2387. 8139024 10.1128/jvi.68.4.2383-2387.1994 PMC236715

[55] Kim JI, Ju WK, Choi JH, Choi E, Carp RI, et al (1999) Expression of cytokine genes and increased nuclear factor-kappa B activity in the brains of scrapie-infected mice. Brain Res Mol Brain Res 73: 17–27. 10581394 10.1016/s0169-328x(99)00229-6

[56] Brown AR, Webb J, Rebus S, Walker R, Williams A, et al (2003) Inducible cytokine gene expression in the brain in the ME7/CV mouse model of scrapie is highly restricted, is at a strikingly low level relative to the degree of gliosis and occurs only late in disease. J Gen Virol 84: 2605–2611. 12917482 10.1099/vir.0.19137-0

[57] Schultz J, Schwarz A, Neidhold S, Burwinkel M, Riemer C, et al (2004) Role of interleukin-1 in prion disease-associated astrocyte activation. Am J Pathol 165: 671–678. 15277240 10.1016/S0002-9440(10)63331-7 PMC1618583

[58] Marcos-Carcavilla A, Calvo JH, Gonzalez C, Moazami-Goudarzi K, Laurent P, et al (2007) IL-1 family members as candidate genes modulating scrapie susceptibility in sheep: localization, partial characterization, and expression. Mamm Genome 18: 53–63. 17242860 10.1007/s00335-006-0095-6

[59] Baker CA, Martin D, Manuelidis L (2002) Microglia from Creutzfeldt-Jakob disease-infected brains are infectious and show specific mRNA activation profiles. J Virol 76: 10905–10913. 12368333 10.1128/JVI.76.21.10905-10913.2002 PMC136595

[60] Baker CA, Manuelidis L (2003) Unique inflammatory RNA profiles of microglia in Creutzfeldt-Jakob disease. Proc Natl Acad Sci U S A 100: 675–679. 12525699 10.1073/pnas.0237313100 PMC141055

[61] Tamguney G, Giles K, Glidden DV, Lessard P, Wille H, et al (2008) Genes contributing to prion pathogenesis. J Gen Virol 89: 1777–1788. 10.1099/vir.0.2008/001255-0 18559949 PMC2828448

[62] Shi F, Yang Y, Kouadir M, Fu Y, Yang L, et al (2013) Inhibition of phagocytosis and lysosomal acidification suppresses neurotoxic prion peptide-induced NALP3 inflammasome activation in BV2 microglia. J Neuroimmunol 260: 121–125. 10.1016/j.jneuroim.2013.04.016 23680490

[63] McElvania Tekippe E, Allen IC, Hulseberg PD, Sullivan JT, McCann JR, et al (2010) Granuloma formation and host defense in chronic Mycobacterium tuberculosis infection requires PYCARD/ASC but not NLRP3 or caspase-1. PLoS One 5: e12320 10.1371/journal.pone.0012320 20808838 PMC2924896

[64] Mayer-Barber KD, Barber DL, Shenderov K, White SD, Wilson MS, et al (2010) Caspase-1 independent IL-1beta production is critical for host resistance to mycobacterium tuberculosis and does not require TLR signaling in vivo. J Immunol 184: 3326–3330. 10.4049/jimmunol.0904189 20200276 PMC3420351

[65] Aguzzi A, Heikenwalder M, Polymenidou M (2007) Insights into prion strains and neurotoxicity. Nat Rev Mol Cell Biol 8: 552–561. 17585315 10.1038/nrm2204

[66] Tixador P, Herzog L, Reine F, Jaumain E, Chapuis J, et al (2010) The physical relationship between infectivity and prion protein aggregates is strain-dependent. PLoS Pathog 6: e1000859 10.1371/journal.ppat.1000859 20419156 PMC2855332

[67] Ayers JI, Schutt CR, Shikiya RA, Aguzzi A, Kincaid AE, et al (2011) The strain-encoded relationship between PrP replication, stability and processing in neurons is predictive of the incubation period of disease. PLoS Pathog 7: e1001317 10.1371/journal.ppat.1001317 21437239 PMC3060105

[68] Baker CA, Lu ZY, Zaitsev I, Manuelidis L (1999) Microglial activation varies in different models of Creutzfeldt-Jakob disease. J Virol 73: 5089–5097. 10233972 10.1128/jvi.73.6.5089-5097.1999 PMC112554

[69] Puoti G, Giaccone G, Mangieri M, Limido L, Fociani P, et al (2005) Sporadic Creutzfeldt-Jakob disease: the extent of microglia activation is dependent on the biochemical type of PrPSc. J Neuropathol Exp Neurol 64: 902–909. 16215462 10.1097/01.jnen.0000183346.19447.55

[70] Shi Q, Xie WL, Zhang B, Chen LN, Xu Y, et al (2013) Brain microglia were activated in sporadic CJD but almost unchanged in fatal familial insomnia and G114V genetic CJD. Virol J 10: 216 10.1186/1743-422X-10-216 23816234 PMC3716817

[71] Prinz F, Schlange T, Asadullah K (2011) Believe it or not: how much can we rely on published data on potential drug targets? Nat Rev Drug Discov 10: 712 10.1038/nrd3439-c1 21892149

