# Supplementary figures and images for: Prion Pathogenesis in the Absence of NLRP3/ASC Inflammasomes
Source: PLoS One. 2015 Feb 11;10(2):e0117208. doi: 10.1371/journal.pone.0117208 (PMC4324825; doi:10.1371/journal.pone.0117208)

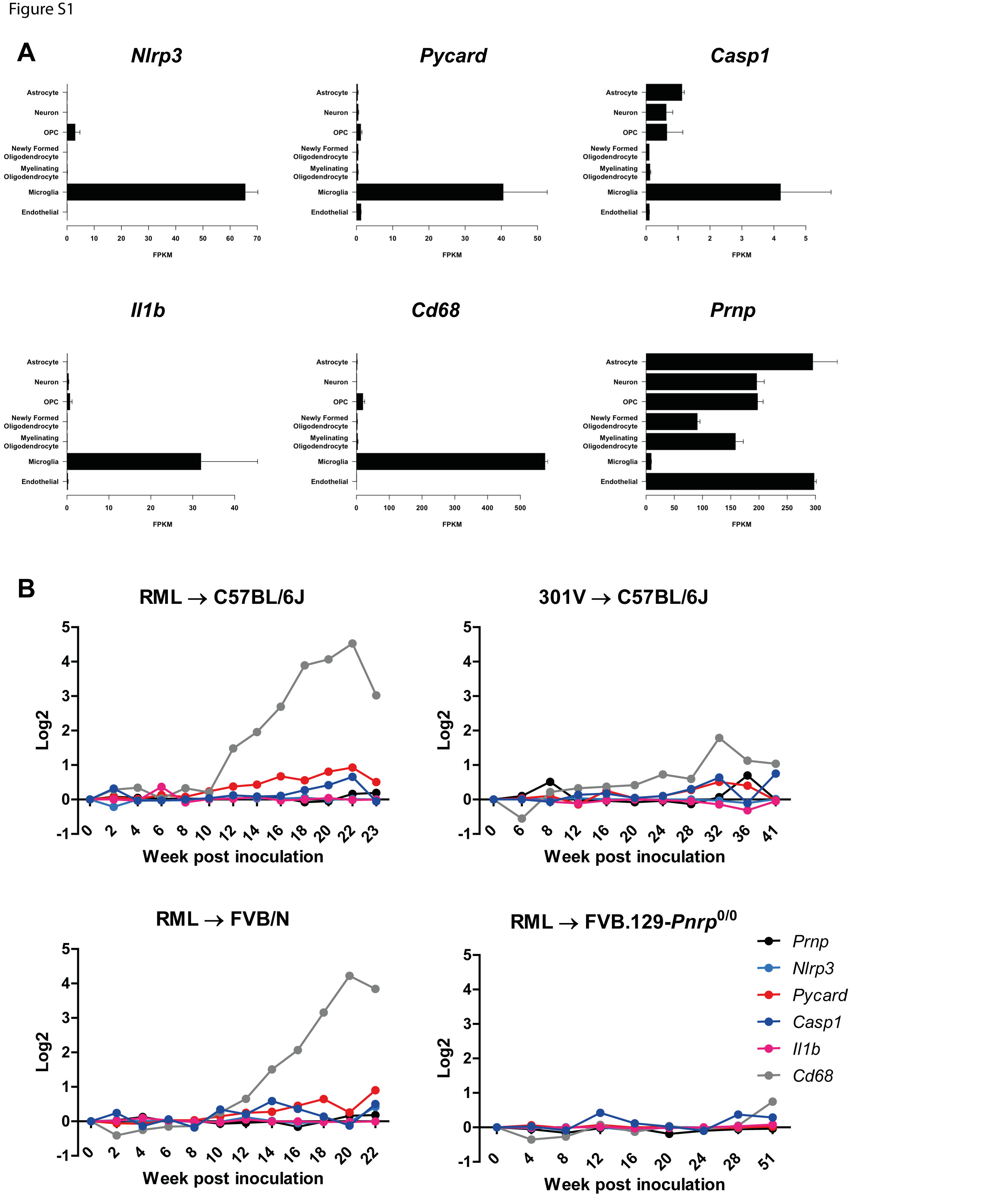

Supplement: S1 Fig — A Expression levels of different NLRP3 inflammasome-related transcripts in different cell types of mouse brain cortex as assessed by RNA-sequencing of acutely purified cell populations. OPC: oligodendrocyte precursor cells; FPKM: fragments per kilobase of transcript per million mapped reads. Based on whole transcriptome profile, it is concluded that OPC cell preparation has 5% of microglia contamination. Scale bars indicate standard deviation. Data and graphs are from Zhang et al. J Neurosci 2014 [24]. B Dynamic gene expression profiles during prion disease development in four mouse strain → prion strain combinations as obtained by microarray analysis. RML→ FVB.129-Prnp0/0 combination serves as control, as these mice lack PrPC and are resistant to prion infection. Data are from Hwang et al. Mol Systm Biol 2009 [25,26]. (TIF) [file pone.0117208.s002.tif]

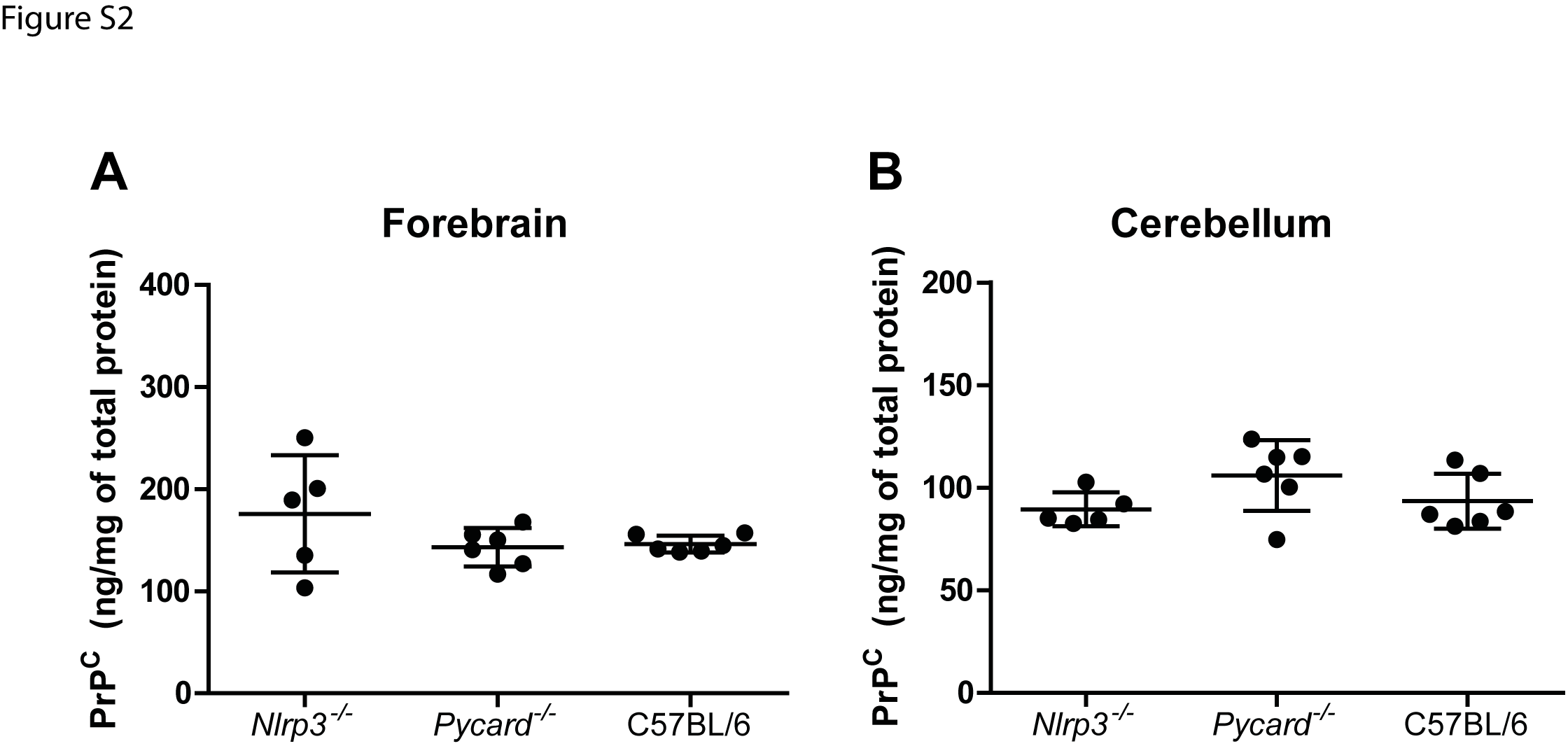

Supplement: S2 Fig — A-B Levels of PrPC in forebrain (A) and cerebellum (B) of Nlrp3-/-, Pycard-/- and C57BL/6 wild-type control mice. Each point denotes one mouse. Mean +/- standard deviation are shown. No significant difference was observed (P = 0.24 in A, P = 0.15 in B, One-way ANOVA). (TIF) [file pone.0117208.s003.tif]

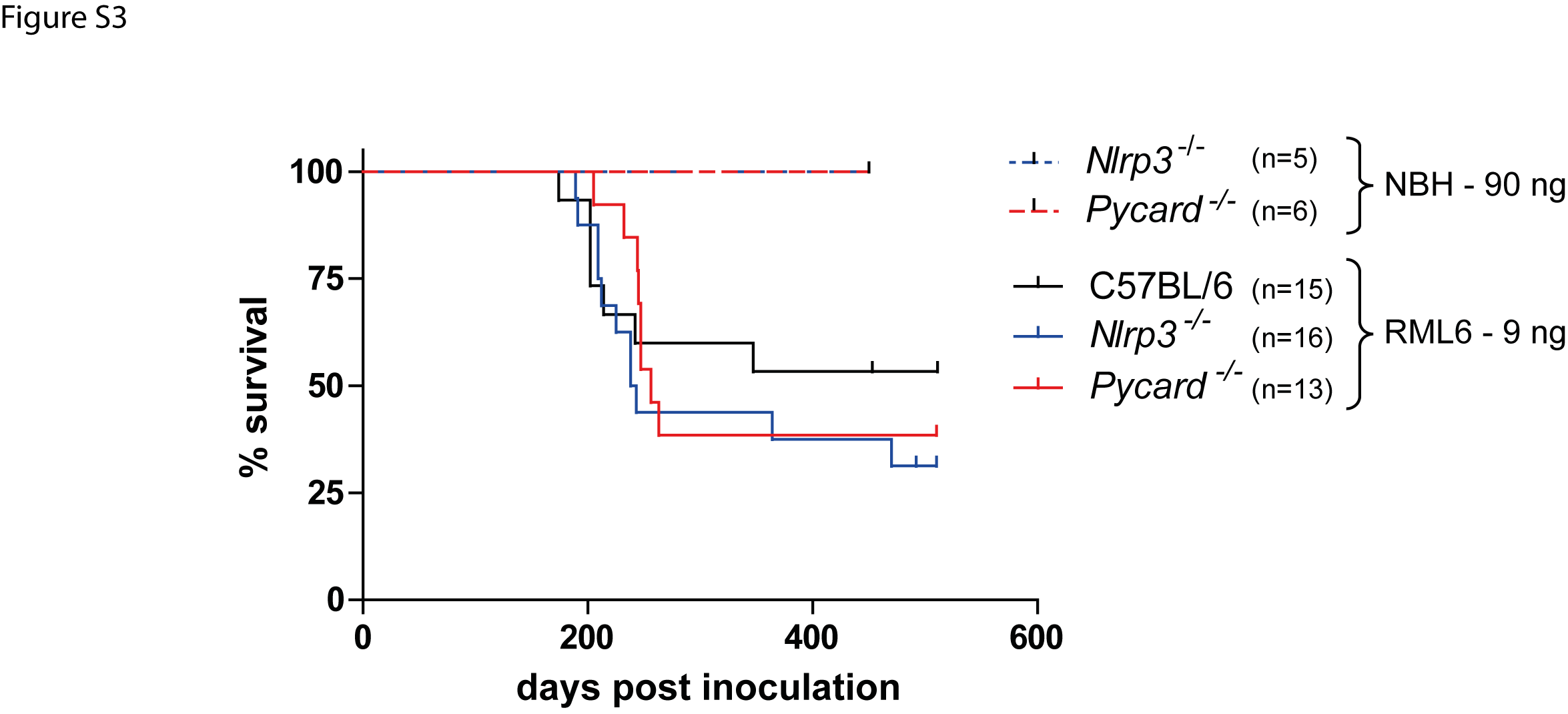

Supplement: S3 Fig — Levels of IL-1β in brains of terminally sick C57BL/6 mice (RML) and control mice injected with non-infectious brain homogenate (NBH). Mice received 30 µg of RML6 or control homogenate. Each point denotes one mouse. Mean +/- standard deviation are shown. No significant difference was observed (P = 0.40, Student’s t test). (TIF) [file pone.0117208.s004.tif]

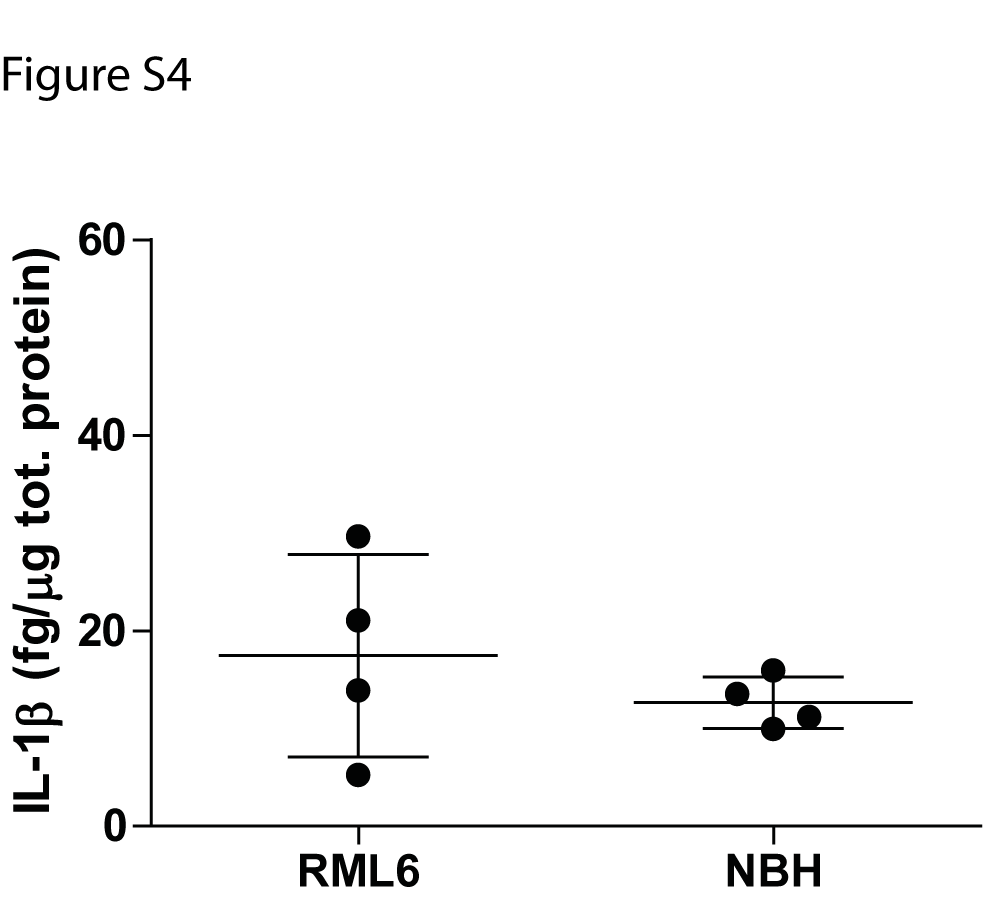

Supplement: S4 Fig — Kaplan-Meier survival plots of Nlrp3-/- (blue line), Pycard-/- (red line) and C57BL/6 wild-type mice (black line) inoculated intracerebrally with 9 ng of RML6. No statistically significant difference among prion-inoculated mice was observed (attack rate and median survival: Nlrp3-/-, 11/16, 241 dpi; Pycard-/- 8/13, 256 dpi; C57BL/6, 7/15, median survival not reached; P = 0.55, log-rank test). Dashed lines indicate mice injected with 90 ng of non-infectious brain homogenates (NBH). For each experimental group, the number of mice is indicated (n). Censored events (ticks) indicate intercurrent deaths not related with prion disease or termination of the experiment. (TIF) [file pone.0117208.s005.tif]

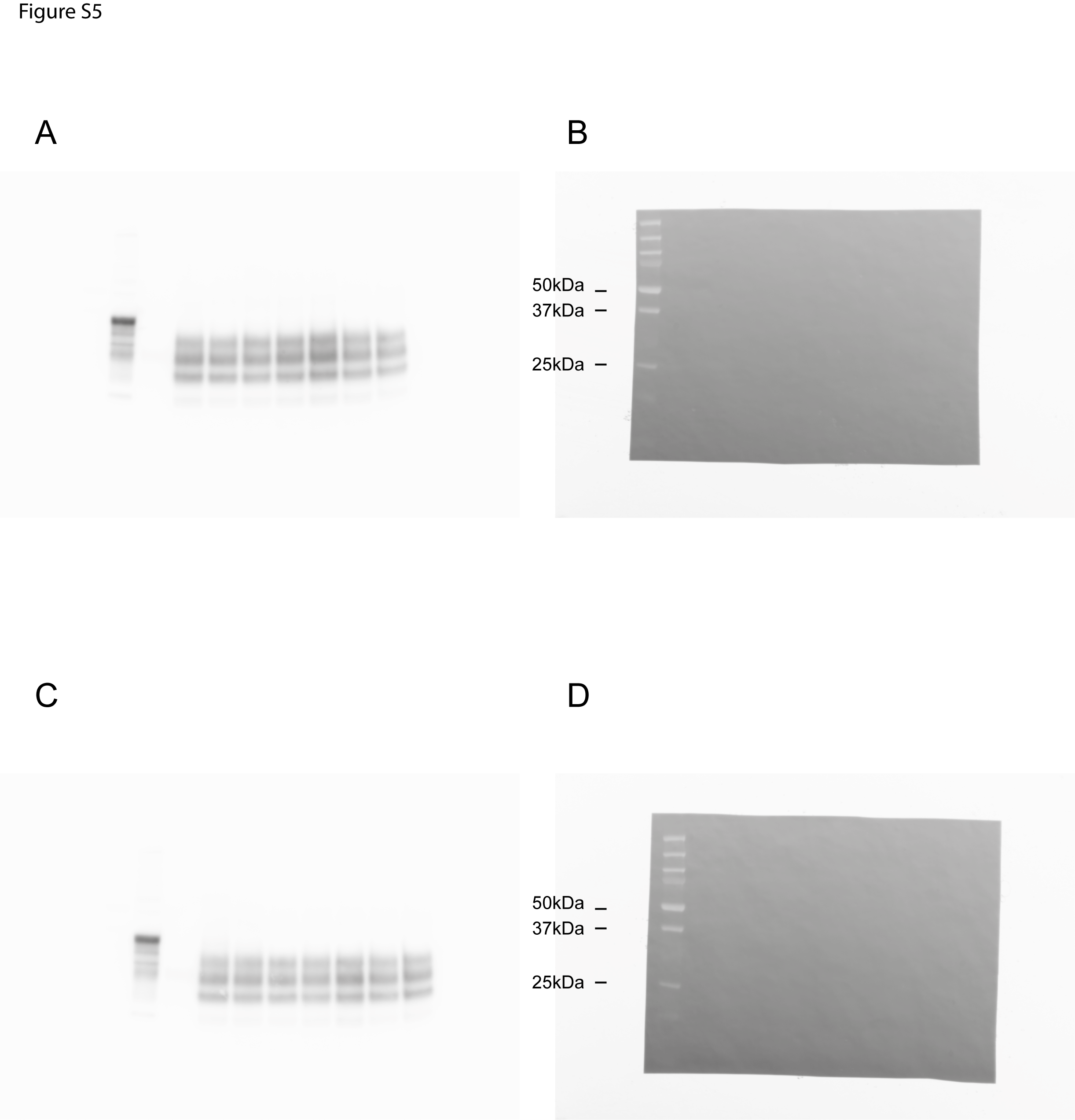

Supplement: S5 Fig — A Original image of Western blot presented in Fig. 1D and the relative image showing molecular size marker in B. C Original image of Western blot presented in Fig. 2D and the relative image showing molecular size marker in D. (TIF) [file pone.0117208.s006.tif]
